# Human iPSC-derived and conventional cancer models in precision oncology: advancing patient-specific therapies from bench to bedside

**DOI:** 10.1186/s13046-026-03694-7

**Published:** 2026-03-30

**Authors:** Tarun Pant, Raman Gulab Brajesh, Billy W. Day, Abhishikt David Solomon, Matea Juric, Jacek Zielonka, Xiaowen Bai

**Affiliations:** 1 Department of Surgery, Division of Pediatric Surgery, Medical College of Wisconsin, 8701 Watertown Plank Road, Milwaukee, WI 53226 USA; 2 Children’s Research Institute, Children’s Wisconsin, Milwaukee, WI 53226 USA; 3 Department of Biomedical Engineering and Bioinformatics, Swami Vivekanand Technical University, Durg, 491107 India; 4 ReNeuroGen LLC, Milwaukee, WI 53122 USA; 5 Adams School of Dentistry, Oral and Craniofacial Biomedicine, University of North Carolina, Chapel Hill, NC 27599 USA; 6 Department of Biophysics, Medical College of Wisconsin, 8701 Watertown Plank Road, Milwaukee, WI 53226 USA; 7 Department of Anesthesiology, Wexner Medical Center, The Ohio State University, 420 West 12Th Avenue, Columbus, OH 43210 USA

**Keywords:** Human induced pluripotent stem cells (hiPSCs), Cancer modeling, Precision oncology, Organoid, AI-derived predictive analytics, Therapeutic resistance, Patient-specific drug response

## Abstract

Targeted cancer therapies, including monoclonal antibodies and lipid nanomedicines, continue to enhance cancer treatment by increasing specificity and prolonging survival. Their therapeutic potential remains, however, limited by tumor heterogeneity, adaptive resistance, and complex microenvironmental factors. Conventional preclinical models, including cancer cell lines, patient-derived xenografts, and organoids, have helped elucidate mechanisms but fail to accurately predict long-term, patient-specific outcomes. This is a methodological gap that still hinders precision oncology, where the main goal is to tailor therapies to individual patients. In this review, we describe conventional in vitro models while focusing on recent developments in the generation and use of human-induced pluripotent stem cell (hiPSC)-derived cancer models. These systems offer distinct opportunities to bridge translational gaps by leveraging oncogenic mutations, providing renewable patient-specific platforms, and combining with lineage tracing, multi-omics, and organ-on-chip technologies. We also evaluate the role of hiPSC-derived models in complementing the existing platforms and discuss current limitations and future development, such as epigenetic mapping, nanoscale testing, and AI-driven analytics, thereby making hiPSC-based cancer models a new, highly promising tool for precision oncology.

## Introduction

Advancements in molecular oncology have made targeted therapies a clinical reality. These include monoclonal antibodies (mAbs), antibody–drug conjugates (ADCs), poly (ADP-ribose) polymerase inhibitors (PARPis), DNA damage response inhibitors (DDRis), and lipid-based nanomedicines [1–6]. A central barrier for further development, including precision oncology, lies in the limitations of current preclinical models, creating a persistent translational gap [7]. Immortalized cancer cell lines have traditionally constituted the foundation for mechanistic research and pharmacological advancement [8]. However, they do not capture intratumoral heterogeneity, accumulate genetic drift over time, and fail to match the biology of patient tumors [7, 9]. Patient-derived xenografts (PDXs) are more physiologically relevant, but are expensive, time-consuming to generate, and limited by species-specific differences that make immune and stromal interactions more difficult [9–11]. Patient-derived organoids (PDOs) have recently opened new opportunities in molecular oncology by preserving some of the genetic and structural features of tumors [12]. Nevertheless, important challenges remain, such as differences in culture, limited scalability, and the inability to fully model tumor-environment interactions, which have made it difficult to connect tumor genotypes with patient responses to treatment [9, 13]. This has led researchers to look for new, genetically accurate, and flexible platforms that can model how tumors change and adapt under treatment pressure.

Human induced pluripotent stem cells (hiPSCs) provide such an opportunity [14, 15]. Due to reprogramming of patient-derived tumor cells into a pluripotent state, hiPSC-based cancer models uniquely preserve oncogenic mutations, maintain clonal diversity, and can be differentiated into tumor-relevant lineages [16–19]. Unlike PDOs, hiPSCs' unique potential to recapitulate the genetic background of individual cancer cells in a pluripotent state represents an untapped opportunity for cancer disease modeling [19]. Additionally, hiPSC-derived cancer models can be engineered to integrate stromal, vascular, and immune components, creating patient-specific systems with broad applications in mechanistic research and drug development [20, 21].

In this review, we critically analyze the strengths and weaknesses of traditional preclinical cancer models and evaluate how the emerging application of hiPSC-based cancer systems provides an opportunity to overcome many of these weaknesses. We point to their use in precision oncology, including patient- or genotype-specific cancer cure, drug resistance, combinatorial drug testing, tissue- and cell-level pharmacological toxicity testing, and immuno-oncology. We also present the outstanding challenges and future directions by which hiPSC-based cancer platforms will continue to influence precision oncology.

## Conventional preclinical models in cancer research

Immortalized cancer cell lines, PDXs, and, more recently, PDOs, as well as organ-on-a-chip models, have been instrumental in cancer research, serving as primary experimental platforms for studying tumor biology, biomarkers, new interventions, and mechanisms driving processes such as cancer cell invasion and metastasis. All these models (Table 1) have contributed uniquely to deciphering the molecular pathways in cell lines, tumor-stroma interactions in PDXs, and patient-specific heterogeneity in PDOs. This section summarizes these models and provides examples of their contribution towards translational oncology.

**Table 1:** Preclinical cancer model usage

Model	Advantages	Limitations	Applications in Cancer Research	References
Immortalized Cancer Cell Lines	Simple to culture, low cost, compatible with high-throughput screening; experimentally tractable and widely used for mechanistic studies	Do not capture intratumoral heterogeneity; can accumulate genetic drift with long-term culture; limited representation of tumor microenvironment and patient tumor biology	Mechanistic studies of oncogenic signaling; drug target discovery; biomarker discovery; early-stage drug screening	[7–9]
Patient-Derived Xenografts (PDXs)	Maintain aspects of tumor architecture and heterogeneity; useful translational surrogates in some contexts	Expensive and time-consuming; low throughput; limited scalability; species-specific differences (mouse stromal/immune context) can complicate immune/stromal biology	Preclinical efficacy testing; biomarker evaluation; resistance modeling	[9–11]
Patient-Derived Organoids (PDOs)	Preserve patient-specific genetic and structural features in 3D; support patient-informed testing (renewable at least short-to-medium term depending on model)	Culture variability and limited scalability; incomplete modeling of tumor-microenvironment interactions; can make it challenging to link genotype to patient treatment response in a standardized way	Precision oncology functionality; drug testing; modeling patient-specific heterogeneity	[9, 12, 13]
Organ-on-a-chip	Recapitulate dynamic tumor microenvironment features (perfusion/flow, mechanical cues, gradients, multicellular organization); can incorporate stromal, endothelial, and immune components; enables real-time functional interrogation	Technically complex and resource-intensive; challenges in standardization and scalability; many workflows still rely on established cell lines or limited primary/autologous samples (not yet broadly renewable/high-throughput patient-specific profiling)	Drug penetration/transport studies; angiogenesis and tumor-vessel interactions; immune-cell recruitment/trafficking and immunotherapy-relevant testing; metastasis and niche-dependent behavior modeling	[22–25]

*PDxs* Patient Derived Xenografts, *PDOs* Patient Derived Organoids

Cancer cell lines, immortalized first in the mid-twentieth century (such as HeLa), are a critical resource in drug discovery, as they can be used in numerous applications, are readily available, and have enabled discoveries that have transformed the field [26]. As an example, the therapeutic efficacy of human epidermal growth factor receptor 2** (**HER2) targeting trastuzumab and inhibition of epidermal growth factor receptor (EGFR) with cetuximab were characterized in breast cancer, feline oral squamous cell carcinoma (FOSCC), and non-small cell lung cancer (NSCLC) cell lines [27–29]. Systematic analysis of cancer cell lines has become more sophisticated in recent years. Domcke et al. have systemically compared genomic copy-number changes, exome mutations, and transcriptomic profiles of 47 ovarian cancer cell lines to 316 high-grade serous ovarian cancer (HGSOC) tumors in the cancer genome atlas (TCGA) and have worked out a quantitative suitability score prioritizing cell lines with TP53 mutations, high-grade serous ovarian cancer (HGSOC)-like copy-number changes, low non-synonymous mutation burden, and no subtype-inappropriate alterations [30]. KURAMOCHI was identified as the best matches to HGSOC copy number alterations (CNAs), whereas popular lines, including OVCAR-3, SK-OV-3, and A2780, did not closely resemble HGSOC tumors mean CNA values [30]. This genomically based framework may bridge the gap between cell lines and primary tumors, enabling more faithful preclinical modeling of HGSOC biology and responses to therapy. Ben-David et al. characterized 27 strains of the conventional MCF7 breast cancer cell line cultured across 7 labs and found that genomic (copy-number variations, structural variants) and transcriptional divergence between samples were high, resulting from passage history and lab-specific culture conditions [31]. These strains showed large differences in their response to a library of 321 anticancer compounds. At least three-quarters of compounds that were strongly active in one strain were inactive in another, indicating a direct connection between culture adaptation, CNV accumulation, and altered therapeutic sensitivity. This approach demonstrates a significant limitation of the use of cancer cell lines and provides a stringent system for measuring and preventing cell line instability, thereby improving reproducibility in drug response assessments [31].

PDXs are an example of methodological breakthroughs that enabled the direct implantation of fresh human tumor tissue into immunodeficient mice, preserving its three-dimensional architecture and recapitulating the genomic characteristics of a patient's tumor [32]. First reports suggested that PDXs maintained the histological features and molecular characteristics of the original tumor, and subsequent work has used PDXs to recapitulate in vivo drug pharmacokinetics, tumor-stroma interactions, and drug resistance [32]. Recently, Pham et al. developed PDX models using 276 pancreato-duodenal and biliary resections (initial P0 engraftment: 59% pancreatic, 86% duodenum, 35% bile duct), and 127 successful PDXs phenotypically (histology) and genotypically faithful to patient tumors using whole exome sequencing (median 76% patient-to-PDX somatic mutation overlaps) and copy-number profiling [33]. These models were heterogeneous in KRAS-MAPK pathway activation, regardless of the presence of KRAS mutations (e.g., EGFR/BRAF changes in KRAS-wild cases), and allowed rational polytherapy testing in PDX-derived organoids (71% success rate) and confirmed their relevance to guide preclinical drug testing in genomically heterogeneous pancreaticobiliary cancers [33]. In another recent example of the progress in PDX methodology, Miura et al. developed an orthotopic tissue fragment xenograft model by directly suturing fragments of fresh pancreatic cancer resection onto mouse pancreatic tails, with rates of distant metastasis to the liver and lungs up to 80%, much higher than with traditional cell suspension injections [34]. These patient-derived orthotopic tissue xenograft (PDOTX) tumors faithfully reproduced clinical features, including invasion of the perineurium, desmoplasia, and cancer-related hypercoagulability (high levels of D-dimer and TAT complexes), as well as increased EMT gene signatures indicative of human disease progression [34]. Complementing orthotopic approaches, Wagner et al. generated subcutaneous PDXs from pancreatic cancer circulating tumor cells (CTCs) and CTC-derived tumor spheres, securing 60% engraftment rates while preserving histological fidelity aberrant ducts, stromal components, and neo vasculature indistinguishable from primary tumors. Metastatic potential was validated using chorioallantoic membrane (CAM) assays, confirming the utility of these liquid biopsy-derived models for studying dissemination and stroma-dependent phenotypes [35]. Collectively, these 2025 studies expand on the subcutaneous PDX cohort of Pham et al. by incorporating orthotopic microenvironment, non-invasive CTC source, and consistently showing over 75% genomic/phenotypic fidelity, maintenance of the KRAS-MAPK signaling, and improved metastatic recapitulation, which facilitates the precision testing of genomic heterogeneous pancreaticobiliary cancers by testing therapy.

Recently, PDOs have emerged as another novel model for cancer research, with the potential to serve as an attractive platform for personalized cancer medicine, as they retain important histologic, genetic, and functional properties of the patient tumor and enable ex vivo drug testing [36]. For example, Vlachogiannis et al. developed a living biobank of 71 PDOs from 53 metastatic colorectal (CRC) and gastroesophageal cancer patients, who were heavily treated in phase I/II clinical trials, with high fidelity to the original tumors in histopathological, whole-exome sequencing, and RNA-seq analyses [37]. Importantly, ex vivo drug-sensitivity testing in these PDOs was highly predictive of patient clinical outcome (unsurpassed sensitivity/specificity of irinotecan/5-FU combinations) and patterns of resistance, and orthotopic xenografts derived from PDOs further confirmed in vivo correlates [37]. The study was a groundbreaking demonstration of PDOs as a clinically predictive culture platform that bridges in vitro scalability and individual tumor biology, enabling precision oncology. Cui et al. have made 28 PDOs out of 32 human pituitary neuroendocrine tumors (PitNET) specimens (87.5% success rate, 95.7% of non-functioning vs. 66.7% of functioning PitNETs) [38]. These PDOs retained parental tumor morphology, subtype-specific neuroendocrine markers (synaptophysin, pituitary hormones, and transcription factors such as TBX19), mutational profiles determined by whole-exome sequencing, and tumor microenvironment heterogeneity, as determined by IHC and sequencing. Subtype-specific responses, e.g., role of ERBB2 expression and ErbB/AMPK pathway enrichment, TGF-β/focal adhesion signatures in the drug response and resistance were found in high-throughput drug screening of 20 clinically relevant agents in aggressive Knosp grade 3/4 PitNET PDOs, confirmed by pharmaco-transcriptomics and two patient cases in which PDO predictions correlated with cabergoline/lapatinib data post-surgery [38]. This groundbreaking PitNET PDO platform extends the limits of the gastrointestinal cancer biobank of Vlachogiannis et al. by demonstrating that PDOs can work with rare neuroendocrine tumors, making them a predictive tool in the precision medicine of refractory PitNETs, where conventional models do not exist [38]. Furthermore, Li et al. generated PDOs and PDO xenografts (PDOX) of pancreatic ductal adenocarcinoma (PDAC) clinical samples, which showed morphological and biological and genomic fidelity as determined by immunohistochemistry, H&E staining, whole-exome sequencing, and RNA sequencing, which revealed uniformity and heterogeneity across models [39]. A 111-drug screen of FDA-approved drugs showed substantial inter-patient variability in sensitivity, with PDO and matched PDOX responses highly concordant with clinical chemotherapeutics. Simultaneously, genome-wide gene expression analysis revealed UGT1A10 as a primary regulator of drug metabolism, whose silencing increased treatment efficacy [39]. An additional PDO-immune cell co-culture model demonstrated immunotherapy-specific cytotoxicity, which confirmed the use of PDOs in treating personalized PDAC. The present research provides strong evidence that molecular profiling and functional drug testing on PDO/PDOX platforms can accurately predict clinical responses and provide a foundation for precision medicine approaches in genomically heterogeneous PDAC patients [39].

Recently, Zhao et al. developed a patient-derived bladder cancer organoid (BC-PDO) biobank that maintained the genetic profile, histopathological characteristics, and tumor microenvironmental heterogeneity of primary tumors, thereby enabling a detailed examination of the driver genes underlying sensitivity to standard-of-care chemotherapeutics, cisplatin, and gemcitabine [40]. Massive drug screening identified genetic changes that predict therapeutic effects, whereas co-culture experiments demonstrated the use of PDOs to study tumor-stroma interactions and drug penetration [40]. This research confirms that BC-PDOs can serve as a preclinical diagnostic tool to elucidate the biology of bladder cancer, predict each patient's prognosis, and guide tailored therapeutic interventions. Despite their translational value, PDX and PDO platforms typically capture only a subset of the dynamic biophysical and transport properties of the in vivo tumor microenvironment, including perfusion-driven drug delivery, oxygen and nutrient gradients, and mechanically regulated invasion. To address these microenvironmental and kinetic limitations, microphysiological “organ-on-a-chip” systems have emerged as complementary platforms that incorporate controlled flow, spatial organization, and multicellular crosstalk into patient-relevant tumor models.

Organ-on-a-chip models have evolved into advanced preclinical devices that can supplement conventional in vitro and in vivo models of tumor biology and therapeutic response [22, 23]. These microphysiological systems integrate microfluidic engineering with three-dimensional cultures to replicate the dynamic characteristics of the tumor microenvironment, including perfusion, mechanical cues, oxygen and nutrient gradients, and multicellular organization [22, 23]. Mechano-responsive, vascularized tumor-on-a-chip devices can be leveraged to interrogate drug penetration, immune cell recruitment and trafficking, angiogenesis, and metastatic behaviors under physiologically relevant flow and mechanical conditions [24]. Notably several platforms incorporated stromal fibroblasts, endothelial networks, and immune components to model tumor microenvironment crosstalk and immune editing. Nevertheless, despite their mechanistic sophistication, organ-on-a-chip systems remain technically complex, resource-intensive, and difficult to replicate or scale across laboratories and indications. Moreover, although recent organ-on-a-chip systems have incorporated patient-derived tumor cells and organoids, many workflows continue to rely on established cell lines. As a result, these systems often lack the ability to support widely renewable, high-throughput, patient-specific genetic profiling at scale [23, 25]. Consequently, while organ-on-a-chip technologies improved physiological relevance, currently they address only a subset of the persistent challenges associated with long-term clonal evolution, intratumoral and stromal heterogeneity, and scalable precision cancer testing [23].

Recent experimental studies in the cancer-on-a-chip models have further demonstrated both their strengths and limitations. A tri-culture of human bone niche cells with breast cancer cells in a well-characterized osteolytic bone-metastasis organ-chip recapitulated hallmark pro-metastatic, bone-destructive behaviors and enabled multi-omics benchmarking against in vivo mouse models, supporting the notion that microphysiological, flow-enabled systems can capture niche-dependent metastatic programs in a controllable and human-relevant format [41]. Immunotherapy-relevant functional tests in reconstructed microenvironments are also being supported by cancer-on-a-chip platforms. As an example, CAR-gd T-cell cytotoxicity was measured under physiologically relevant conditions in a patient-derived glioblastoma organoid-microfluidic system that combined microgravity organoid culture with a perfused chip (the Micro-GRA&FLU platform). This system enabled comparative evaluation of monotherapy and combination regimens, demonstrating that the combination of CAR-gd T cells with PD-1 blockade or TREM2 blockade enhanced antitumor activity in the chip-based microfluidic system, illustrating the utility of tumor-chip platforms for assessing immune-based therapies [42].

Drug transport and penetration phenomena have likewise been interrogated using vascularized tumor-on-a-chip models. The microfluidic, heterotypic tumor model generated vessel-supported tumor constructs with an endothelial network surrounding and demonstrated that a vessel-supported, fibroblast-rich tumor microenvironment was a potent suppressor of paclitaxel activity relative to avascular tumor beads and indicated how vascular and stromal architecture might modulate therapeutic exposure and resistance phenotypes [43]. Moreover, vascularized tumor-on-a-chip models that recapitulate tumor-induced angiogenesis and tumor-vessel interactions allow real-time visualization of endothelial sprouting toward tumor spheroids and of antiangiogenic therapy-induced perturbation of the vascular network, providing a tractable system to study dynamic vascular-tumor crosstalk, which is important for drug delivery and efficacy [44]. In addition, microfluidic compression platforms, which impose controlled solid stress on tumor cells or spheroids have provided a quantitative framework linking biophysical loading to altered cellular deformation and viscoelastic properties, as well as associate these mechanical phenotypes with more aggressive tumor behavior, thereby experimentally correlating solid stress with tumor progression [45]. Together, these studies demonstrate the power of cancer-on-a-chip technologies to test the drug response and to study the mechanisms of metastasis, and immune response. However, challenges related to standardization, throughput, and the availability of broadly renewable patient-specific cellular sources persist [41–45].

Collectively, these cancer cell lines, PDXs, PDOs, and organ-on-a-chip models have provided mechanistic insights and experimental systems that have facilitated the development of modern targeted therapies. They continue to significantly contribute to translational oncology, and the following section will discuss the extent to which the ADCs, PARPis, DDR inhibitors, lipid nanomedicines, and other cancer-targeting strategies have been tested in these conventional preclinical models, with the existing limitations and challenges identified.

## Conventional preclinical models in targeted therapy development: applications and challenges

As noted above, conventional preclinical cancer models including immortalized cell lines, PDXs, and PDO-based systems have been central to development of targeted therapy by enabling mechanistic discoveries, biomarker identification, and preclinical efficacy testing. At the same time, these platforms exhibit recurring limitations that contribute to the translational gap, including incomplete representation of tumor microenvironment complexity, restricted patient specificity and genetic diversity, and inconsistent predictive clinical therapeutic response across tumor types.

### Applications of conventional preclinical models in targeted cancer therapy development

**Fig. 1:**
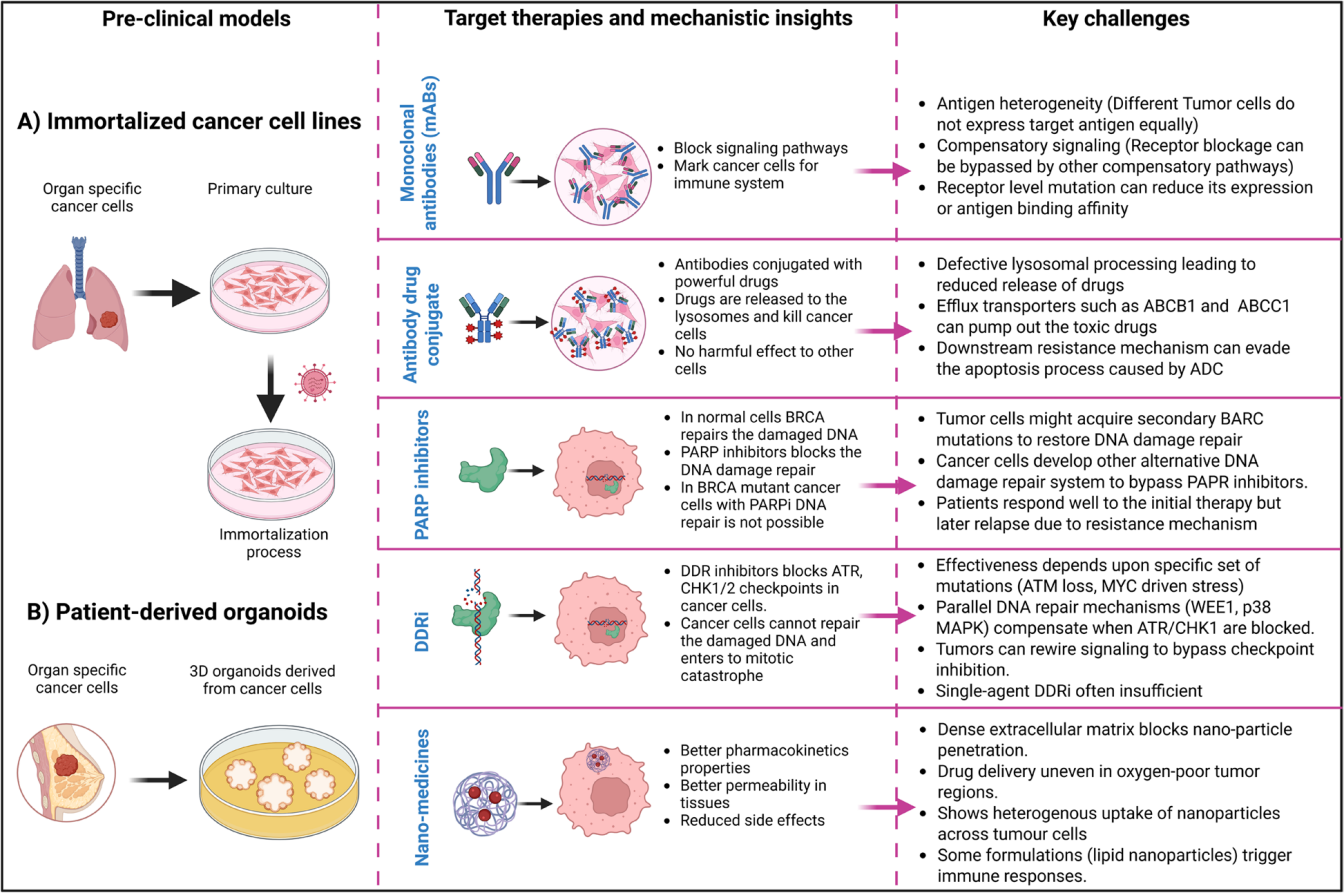
Mechanistic insights and emerging challenges revealed by preclinical models. ADC: Antibody Drug Conjugate; ABCB1: ATP-binding cassette subfamily B member 1; ABCC1: ATP-binding cassette subfamily C member 1; ATM: Ataxia-Telangiectasia Mutated; ATR: Ataxia Telangiectasia and Rad3-related protein; CHK ½: Checkpoint Kinase 1 and 2; BRCA: Breast Cancer gene; DDR: DNA Damage Response; and PARP: Poly ADP Ribose Polymerase

Advances in molecular oncology have accelerated the development of therapies that selectively target cancerous cells while sparing normal tissues. Of these, mAbs, ADCs, adoptive cell therapy (ACT, e.g., CAR T-cell therapy), PARPis, DDRis, and lipid nanomedicine formulations represent major classes of targeted cancer therapies currently shaping clinical practice (Fig. 1). Preclinical model systems such as immortalized cancer cell lines, PDXs, and PDOs have been the major platforms used for defining drug mechanisms, identifying predictive biomarkers, and serving as preclinical translational surrogates before clinical testing. Mechanistic insights and overview of targeted cancer therapy classes across distinct conventional preclinical models are summarized in Tables 2 and 3 and described below.

**Table 2:** Mechanistic insights across conventional preclinical cancer models

Model	Tumor Biology & Pathway Dependence	Intratumoral Heterogeneity & Clonal Evolution	Tumor microenvironment interactions	Drug Transport/Penetration & Dynamic Gradients	Biomechanics & Solid Stress	Practicality: Scalability, Standardization & Throughput	References
Immortalized Cancer Cell Lines	Enable controlled interrogation of oncogenic signaling and drug-target interactions; commonly utilized for mechanistic experiments	Can show genomic and transcriptional differences caused by culture or passage; medication responses can vary widely among strains	Limited microenvironment representation	Limited ability to model perfusion-driven transport and gradients	Standard cultures do not simulate solid stress	Easy to get and scale up; lab-specific variables can affect its performance	[26–29, 31]
Patient-Derived Xenografts (PDXs)	Maintain tumor architecture and patient genomic characteristics to facilitate in vivo studies	Capture patient tumor heterogeneity and facilitate in vivo resistance investigations, enhancing metastatic recapitulation in orthotopic and CTC-derived methodologies	Facilitate in vivo tumor-stroma interactions and resistance phenotypes within a living host context	Provide in vivo delivery context but lower experimental control and throughput	Tissue mechanics occur in vivo, but it is difficult to isolate the mechanical variables that cause them	Resource-intensive; throughput constraints implied by in vivo nature	[32–35, 37–40]
Patient-Derived Organoids (PDOs)	Maintain histological, genetic, and functional characteristics while facilitating ex vivo functional testing in patient-informed settings	Drug screening demonstrates inter-patient variability	Co-culture methodologies can simulate tumor-stroma interactions and functional cytotoxicity; certain platforms indicate the preservation of microenvironmental heterogeneity	Ex vivo testing; certain co-culture/penetration experiments are feasible, but they do not inherently replicate perfusion/mechanical regimes	3D structure is helpful, but controlled mechanical loading is not very effective	Can help with drug screens and biobanks, although the success rates and scalability depend on the type of tumor and the protocol	[36–40]
Organ-on-a-chip	Adds regulated flow/mechanics and multicellular organization for pathway behavior that is relevant to humans under dynamic conditions	Improves physiological fidelity but doesn't completely solve long-term clonal evolution and scalable heterogeneity modeling	Includes stromal fibroblasts, endothelial networks, and immune components to show how crosstalk and immune editing function as blood flows across them	Explicitly designed to model perfusion/gradients; primary studies show vascular/stromal architecture impacts paclitaxel activity and allow angiogenesis visualization	Microfluidic compression platforms provide regulated solid stress and connect deformation/viscoelastic behavior to aggressive phenotypes	It is technically challenging and resource-intensive, and it is still hard to standardize and scale across labs and indications	[22–25, 43–45]

*PDxs* Patient Derived Xenografts, *PDOs* Patient Derived Organoids

**Table 3:** Overview of targeted cancer therapy classes and mechanistic insights from conventional preclinical models

Therapy Class	Mechanism of Action	Representative Agents	Key Limitations	References
Monoclonal Antibodies (mAbs)	Inhibit oncogenic activity and stimulate immune effector mechanisms	Trastuzumab (HER2), Cetuximab (EGFR), Bevacizumab (VEGF)	antigen heterogeneity and adaptive resistance in solid tumors	[46–52]
Antibody–Drug Conjugates (ADCs)	Combine mAbs with cytotoxic payloads; internalization and lysosomal release induce apoptosis	Trastuzumab deruxtecan (HER2 + breast cancer), Sacituzumab govitecan (Trop-2 + TNBC)	Overlapping DNA repair pathways increase off-target toxicity risk; pathway redundancy complicates translation	[53–56]
Adoptive Cell Therapy	Engineered immune cells (e.g., CAR T) recognize tumor antigens and mediate cytotoxicity		Incompletely recapitulate patient-specific immune context, clonal evolution under immune pressure, and spatial barriers to immune access especially for solid tumors	[53–56]
DNA Damage Response Inhibitors (DDRis)	Target ATR, ATM, CHK1/2 to exacerbate replication stress and DNA repair deficiencies	ATR inhibitors (ceralasertib), CHK1/2 inhibitors (prexasertib)	Narrow therapeutic window; compensatory signaling; modest efficacy in trials	[53]
Poly(ADP-Ribose) Polymerase Inhibitors (PARPis)	Exploit synthetic lethality; inhibit DNA single-strand break repair, leading to replication fork collapse in HR-deficient tumors	Olaparib, Niraparib, Talazoparib	Acquired resistance via secondary BRCA mutations, replication fork stabilization, alternative DNA repair pathways	[57–66]
Lipid Nanomedicines	Liposomal or nanoparticle carriers improve drug stability, delivery, and tumor targeting; reduce systemic toxicity	Liposomal doxorubicin, lipid nanoparticles for siRNA/mRNA delivery	Variable intratumoral uptake; microenvironmental barriers; patient-specific differences in metabolism and endocytosis	[6, 67, 68]

*ADCs* Antibody–drug conjugates, *ACT* Adoptive Cell Therapy, *ATR* Ataxia telangiectasia mutated and Rad3-related, *ATM* Ataxia Telangiectasia Mutated kinase, *BRCA* Breast Cancer gene, *CART* Chimeric Antigen Receptor Therapy, *CHK1/2* Checkpoint Kinase 1 and 2, *DDR* DNA Damage Response Inhibitors, *EGFR* Epidermal growth factor receptor, *HER2* Human Epidermal growth factor Receptor 2, *mRNA* Messenger RNAs, *mAbs* Monoclonal antibodies, *PARPis* Poly (ADP-ribose) polymerase inhibitors, *siRNA* Small interfering RNA, *TROP-2* Tumor-associated calcium signal transducer 2, *TNBC* Triple-negative breast cancer, *VEGF* Vascular growth factor receptor

Cell line- and PDO-based research demonstrated that mAbs can inhibit oncogenic activity and stimulate immune effector mechanisms, and this resulted in the development of new drugs such as trastuzumab, cetuximab, and bevacizumab [46–51]. These models also predicted the main response limitations, such as antigen heterogeneity and adaptive resistance in solid tumors [52]. On the same note, these preclinical systems played a key role in determining ADC activity, showing that therapeutic efficacy is not solely reliant on antigen binding but also on intracellular trafficking and lysosomal processing of cytotoxic payloads [69–72].

ACT, including chimeric antigen receptor (CAR) T-cell therapy, represents another major category of targeted cancer therapy in which engineered immune cells recognize tumor-associated antigens and mediate tumor cell cytotoxicity and clearance [53]. Conventional preclinical models have been instrumental in defining antigen selection and target density thresholds, optimizing CAR designs, elucidating immune effect mechanisms, and identifying resistance drivers such as antigen loss/heterogeneity, impaired trafficking and infiltration, and immunosuppressive signals withing tumor-microenvironment [54, 55]. Xenograft systems, particularly those incorporating human tumor cells and, when feasible, human immune components, have supported proof-of-concept testing of CAR constructs and rational combination strategies (e.g., checkpoint blockade, cytokine modulation, or stromal-targeting approaches) [56]. These platforms often, however, incompletely recapitulate patient-specific immune context, clonal evolution under immune pressure, and spatial barriers to immune cell access that are central to ACT performance in solid tumors, underscoring the need for improved, more human-relevant, scalable, and patient-informed model systems.

The more traditional preclinical models have also played key roles in mapping the susceptibility to DNA damage response (DDR)-targeting agents. The studies with cell lines and xenografts demonstrated the synthetic lethality principles of PARP inhibitor therapy in tumors with BRCA1 (BReast CAncer gene 1) and BRCA2 (BReast CAncer gene 2) deficits and were instrumental in the development of DDR-inhibiting strategies targeting the ataxia telangiectasia and rad3-related (ATR), ataxia telangiectasia mutated (ATM), and checkpoint kinase** (**CHK1/2) pathways [57–65]. The DDRis recognize and kill proliferative cancer cells through inhibiting key proteins (e.g., PARP, ATR, and ATM) that induce repair and replication stress in rapidly proliferating tumor cells. Initial research on DDR inhibitors has shown effectiveness in various types of cancer, and further research is currently underway. Their clinical application, however, is restricted by a narrow therapeutic index and overlapping DNA repair pathways that increase the risk of off-target toxicity [66]. These platforms facilitated combinatorial strategy screening and resistance mechanism identification but also identified pathway redundancy and toxicity limits that complicate clinical translation.

Parallel lipid-based nanomedicine systems have been tested in cell and organoid models, where they exhibited improved drug stability, as well as enhanced tissue delivery properties, and have been clinically approved as liposomal doxorubicin [6, 67, 68]. Nevertheless, preclinical trials consistently indicate that drug uptake and therapeutic response are highly variable and are strongly influenced by both tumor-intrinsic and microenvironmental factors.

Collectively, cancer cell lines, PDXs, and PDOs have greatly contributed to the mechanistic and translational basis of contemporary targeted cancer therapies, including antibody-based agents, adoptive cell therapies, and small-molecule and nanomedicine approaches. At the same time, drug response and resistance trajectories often differ across model types and across tumors, reflecting heterogeneity and incomplete representation of key microenvironmental and evolutionary features. Consequently, the predictive value of conventional models for patient-specific outcomes can be variable, underscoring the need for next-generation, patient-relevant platforms, outlined in the sections below.

### Challenges and translational gaps of conventional models

**Fig. 2:**
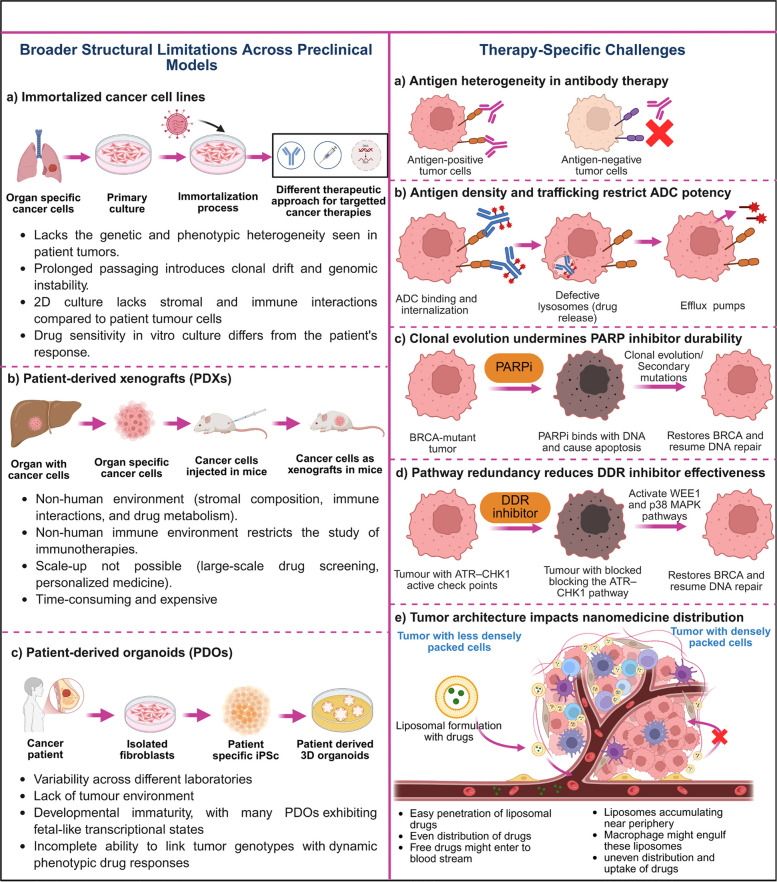
Challenges of current preclinical models. ADC: Antibody Drug Conjugate; ATR: Ataxia Telangiectasia Rad3-Related Kinase; BRCA: Breast Cancer gene; CHK1: Checkpoint Kinase 1; DDR: DNA Damage Response; iPSCs: Induced Pluripotent Stem Cells; PDxs: Patient Derived Xenografts; PDOs: Patient Derived Organoids; PARP: Poly ADP Ribose Polymerase; PARPi: Poly ADP Ribose Polymerase Inhibitors; and P38 MAPK: p38 Mitogen-Activated Protein Kinase

Cell lines- and PDOs-based studies not only have enabled the identification of many fundamental mechanisms of targeted therapies but have also revealed therapy-specific obstacles to clinical durability [73, 74]. The identified challenges include intratumor heterogeneity, clonal evolution of targets under drug pressure, variability in antigen density and localization, and alterations in tumor architecture that change tissue distribution (Fig. 2). For example, Minussi et al. showed that single-cell copy-number analysis of triple-negative breast cancers (TNBCs) and matched cell lines reveals that breast tumors harbor 7–22 subclones organized into a few superclones and continue to acquire subclonal copy number aberrations (CNAs) during primary tumor growth, thereby preserving a large reservoir of genomic diversity. Experimental subcloning of MDA-MB-231 demonstrated that single "isogenic" clones rapidly diversify their genomes, with subclonal CNAs driving gene dosage and transcriptomic variability [75]. Additionally, Kim et al.’s single-cell DNA/RNA sequencing of 20 TNBC patients pre/post-neoadjuvant chemotherapy (NAC) identified two response classes: clonal extinction (10 patients) vs. persistence (10 patients), with 8 analyzed at single-cell resolution (~ 900 DNA, 6,862 RNA profiles). In persistence cases, resistant genotypes (mutations/CNAs) were pre-existing and adaptively selected by NAC, while chemoresistant phenotypes (EMT, hypoxia, ECM degradation, AKT serine/threonine kinase 1 (AKT1) and mechanistic target of rapamycin (mTOR) signatures emerged via transcriptional reprogramming. Single-nucleus copy-number analysis confirmed minor resistant subclones expanded post-NAC (e.g., P14: 7.7% to 71.8%), with no recurrent resistant CNAs but convergent transcriptional programs linked to poor survival in METABRIC cohorts [76]. This persistent intratumor heterogeneity and rapid subclonal evolution, even in cell lines traditionally viewed as monoclonal, represent fundamental barriers to achieving durable responses with targeted therapies, underscoring the urgent need for preclinical platforms that can capture and model ongoing genomic diversification under therapeutic selection.

Recently, Alec et al. performed integrative clustering of 134 TCGA TNBC tumors using gene expression, miRNA, and CNV data, identifying three molecularly distinct subtypes with divergent biology and clinical outcomes [77]. Cluster 1 (31%) showed the highest genomic instability (168 amplified genes), Cluster 2 (28%) the lowest (28 genes), and Cluster 3 (41%) intermediate (126 genes), with unique mutational burdens and signature CNVs distinguishing immune-hot (Cluster 1) vs stroma-rich (Cluster 3) phenotypes. EGFR, FGFR2, and MYC amplifications varied significantly across clusters, directly affecting the applicability of targeted therapy. Cluster-specific miRNA-gene regulatory networks revealed subtype-defining expression programs, with survival differences (Cluster 2 best prognosis) [77]. This demonstrates how multi-omics intratumor heterogeneity creates antigen/protein expression variability that challenges uniform therapeutic targeting across TNBC patients, supporting the "variability in antigen density" challenge while quantifying the genomic basis for differential drug sensitivity.

Tumor architecture further complicates therapy delivery as single-cell profiling reveals proliferative, myofibroblastic, and inflammatory subtypes that remodel tumor architecture and restrict effector access to heterogeneous tumor subclones. Recently, Wu et al. integrated single-cell RNA-seq (12 TNBC samples, 67,386 cells) with bulk RNA-seq (465 TCGA TNBCs) to dissect cancer-associated fibroblast (CAF) heterogeneity, identifying three functionally distinct subtypes including proliferative CAFs (prCAFs) enriched in cell cycle/S-phase genes, myofibroblastic CAFs (myCAFs) expressing ACTA2/α-SMA with ECM remodeling signatures, and inflammatory CAFs (iCAFs) secreting cytokines like IL6/IL8 [78]. These subtypes showed spatial organization with myCAFs forming perivascular barriers and prCAFs co-localizing with hypoxic tumor cores, directly linking fibroblast diversity to altered tumor architecture. Bulk deconvolution confirmed subtype abundance predicts stromal scores and poor prognosis, with myCAF-high tumors exhibiting dense collagen deposition that restricts antibody/drug penetration to antigen-variable tumor subclones. Trajectory analysis revealed CAF plasticity (myCAF to prCAF under hypoxia), explaining dynamic resistance to therapies requiring drug tissue delivery [78].

Based on the studies mentioned above, it is crucial to develop new experimental models built on renewable, genetically defined, and quality-controlled cells that can reproduce intra-tumor heterogeneity and evolution, as well as interactions with microenvironmental components. One way to address these gaps is to use hiPSC-based cancer models.

## Next-generation cancer models: hiPSC-derived platforms

hiPSC-derived cancer models have emerged as next-generation models aimed at overcoming some of the shortcomings of conventional models by reprogramming somatic cells, including skin fibroblasts, urine-derived cells, or peripheral blood cells, with defined transcription factors, most commonly OCT4, SOX2, KLF4, and c-MYC. The resulting hiPSC lines are genetically identical to the donor allowing patient-specific modeling. Contrary to finite primary cultures, hiPSCs can be cultured virtually infinitely in vitro, permitting reproducible and scalable in vitro experimentation over time and location. hiPSCs also can differentiate to all three germ lineage derivatives, resulting in production of lineage-specific cell types, such as neuronal, epithelial, hematopoietic and cardiac cells, and complex forms of tissue, including brain organoids [79–82]. This differentiation versatility also enables the generation of hiPSC-derived epithelial cells and tissue-specific organoid systems that can be leveraged to model lineage-relevant tumor contexts, engineered microenvironments, and therapy response phenotypes in three-dimensional formats.

**Fig. 3:**
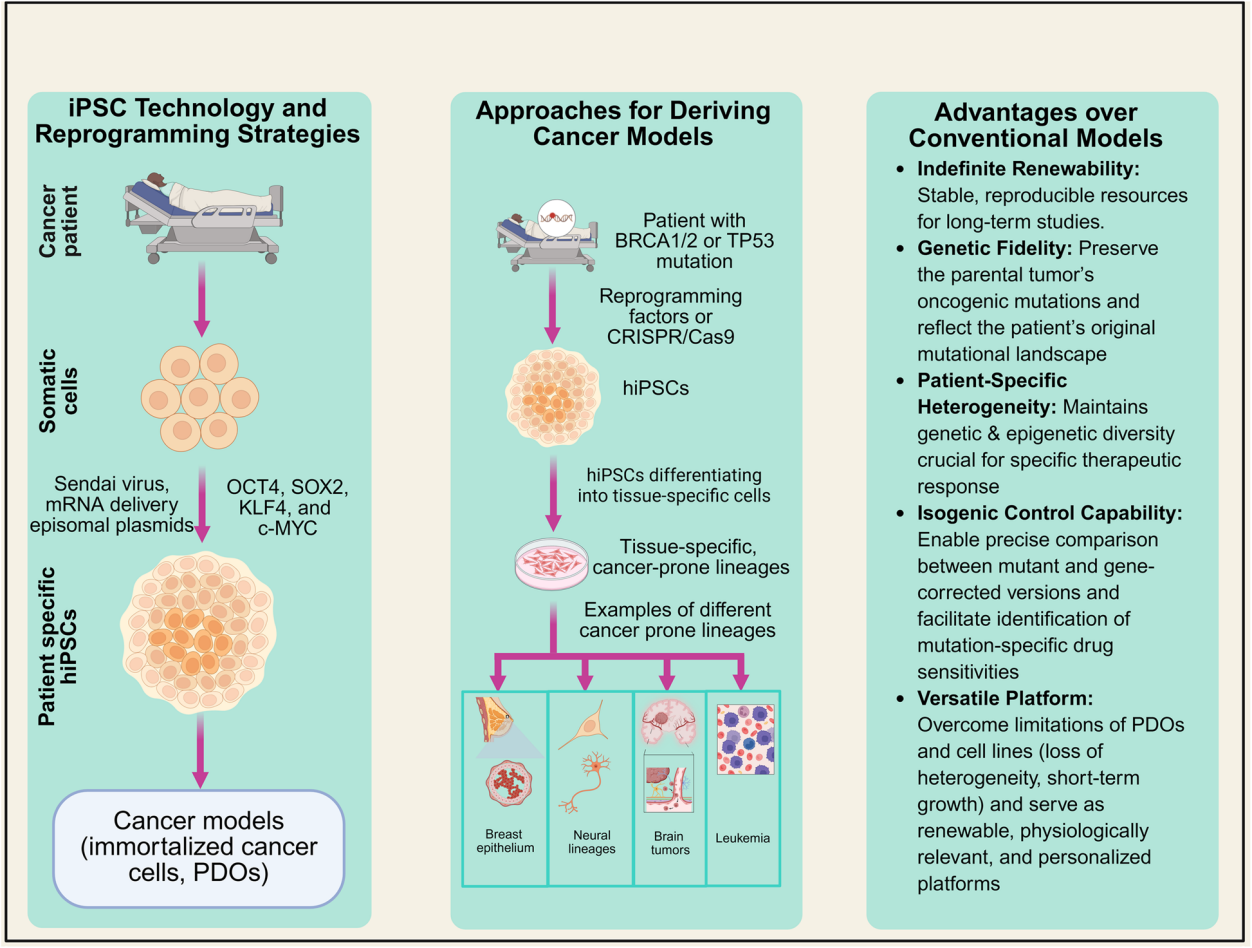
Reprogramming strategies and approaches for deriving hiPSC based cancer models: BRCA: Breast Cancer gene; Cas9: CRISPR-associated protein 9; CRISPR: Clustered Regularly Interspaced Short Palindromic Repeats; C-MYC: Cellular Myelocytomatosis oncogene; hiPSCs: Human induced pluripotent stem cells; KLF4: Kruppel-like factor 4; OCT4: Octamer-binding transcription factor 4; SOX2: SRY-box transcription factor 2; TP53: Tumor Protein 53

hiPSC-based tumor models could be set-up by means of patient-specific hiPSCs or genetically engineered hiPSCs, which are then differentiated into tumor-relevant cell-of-origin lineages. These systems offer controlled and renewable platforms to investigate cancer phenotypes, such as proliferation, migration, and invasion as well as therapeutic response or resistance (Fig. 3). Notably, hiPSC-based cancer models maintain patient-specific genetics and oncogenic mutations engineered and provide experimental control and scalability [20]. The hiPSC platforms perform better than PDOs in simulating tumor stage-specific and lineage-specific drug response in conditions of complete control. With their genetic fidelity, renewability, and differentiation versatility, hiPSC-based cancer models are a promising architecture for advancing the development of precision oncology and enhancing translational predictability in targeted therapy testing. The subsections below outline the basic concepts of hiPSC production, key approaches to construct hiPSC-based cancer models, and engineered hiPSC-based cancer models through genome engineering.

### hiPSC reprogramming and optimization strategies for cancer modeling

The hiPSC technology was first developed by reprogramming somatic cells to a pluripotent state using transcription factors such as OCT4, SOX2, KLF4, and c-MYC [83]. Several non-integrating mechanisms to improve the efficiency and safety of reprogramming have been identified, including episomal plasmids, mRNA delivery systems, Sendai virus vectors, and small-molecule-based approaches [84–89].

Subsequently, optimization of the reprogramming conditions further enhanced maintenance of tumor-specific properties. Hypoxic culture (1–5% O_2_) was discovered to maximize reprogramming and stabilized the HIF-regulated networks of pluripotency, reducing oxidative stress-mediated apoptosis; notably, hypoxia also maintained cancer-specific epigenetic signatures that are typically lost under normoxia, leading to hiPSC derivatives with enhanced fidelity to parental tumor lineage [90–93]. Parallel strategies focused on matching metabolism to the glycolytic state common to cancer cells and pluripotent stem cells, including augmenting glucose supply, supplementing pyruvate, inhibiting oxidative phosphorylation, and stimulating glycolytic regulators such as pyruvate kinase M2 (PKM2) and lactate dehydrogenase-A (LDHA) [88, 94]. These metabolic manipulations maintained epigenetic characteristics associated with oncogenic metabolism and facilitated a more accurate re-emergence of malignant traits during differentiation. A third notable optimization was transient silencing of the p53 pathway, which in addition to enhancing reprogramming efficiency by preventing apoptosis and senescence, allowed maintaining key genomic changes and DNA repair abnormalities specific to tumor cells [95, 96]. A combination of hypoxia, glycolytic support, and p53 inhibition made hiPSC-based cancer modeling a powerful and reproducible method that makes it possible to generate pluripotent intermediates that retain oncogenic mutations, epigenetic identity and malignant transcriptional states needed to accurately model cancer.

### Tumor-derived hiPSC cancer models

In cancer research field, hiPSCs can be also derived from patient-derived tumor cells [97]. In hiPSC-based cancer models, malignant cells are first reprogrammed into a pluripotent state and subsequently differentiated to recapitulate the cellular identity and phenotypic features of the original cancer [20, 21]. This strategy preserves important oncogenic mutations and overcomes the genetic drift and low proliferation capacity of immortalized cell lines and PDOs, respectively [20, 21]. Initial efforts to convert primary tumor cells into a pluripotent state were valuable proof-of-concept studies demonstrating that cancer cells could re-acquire a pluripotent state without losing oncogenic mutations including TP53, KRAS, BCR-ABL, PTPN11, PTEN, and CML [17, 98, 99]. Melanoma, breast cancer, and leukemia studies indicated that reprogrammed tumor-derived iPSCs could be differentiate back into lineage-specific cancer cell phenotypes, during which they recovered malignant phenotypes, including aberrant proliferation, infiltrative potential, and parental tumor transcriptomic signatures [98].

### Engineered hiPSC cancer models using genome editing

Several strategies have been developed to modify hiPSC technology for oncology applications. Pioneering work by Lee et al. generated patient-derived iPSCs from Li-Fraumeni syndrome (LFS) fibroblasts harboring heterozygous TP53 G245D, differentiating them into osteoblasts (OBs) that exhibited defective osteogenic differentiation, transformed piled-up morphology, cell cycle/mitosis enrichment, and tumor-initiating capacity in chick CAM and mouse xenograft models, with LFS-OB transcriptional signatures correlating to poor osteosarcoma prognosis. In this model, mutant p53 repressed the imprinted lncRNA H19, whose restoration reduced tumorigenicity via upregulation of DECORIN, whereas p53 knockdown rescued osteogenic defects and attenuated malignant features [100]. In addition to the transcriptional and genomic effects of mutant p53, recent hiPSC-based studies also point to an additional transcriptionally regulative role for the m6A epitranscriptomic machinery. Xu et al. demonstrated that physical interactions between mutant p53 and SVIL recruit the H3K4me3 methyltransferase MLL1 (KMT2A) and activate transcription of the m6A reader YTHDF2 by increasing H3K4me3 at the YTHDF2 promoter in LFS hiPSC-derived astrocytes, a cell-of-origin model for glioma [101]. The mutant p53-SVIL-MLL1 axis is linked to reduced global m6A mRNA methylation and abnormal upregulation of the YTHDF2, which in turn leads to increased YTHDF2-dependent decay of m6A-marked transcripts and decreased expression of multiple tumor-suppressive targets, including CDKN2B and SPOCK2, thereby driving oncogenic reprogramming in LFS astrocytes. Notably, genetic depletion of YTHDF2 or pharmacologic inhibition of the MLL1 complex had a significant effect in attenuating mutant p53-driven neoplastic phenotypes, such as colony growth in soft agar and other transformation-related readouts [101]. Collectively, these findings indicate that mutant p53 has the potential to couple chromatin remodeling to m6A-dependent RNA fate control, underscoring that hiPSC-based platforms can reveal post-transcriptional programs that collaborate with canonical mutant p53 gain-of-function mechanisms during neoplastic transformation [101].

Building on this, Orsi et al. established two high-quality hiPSC lines (HDF108 from a 27-year-old male, HDF109 from a 38-year-old female) from healthy donor skin fibroblasts using non-integrating Sendai virus vectors (CytoTune 2.0; KOSM, c-MYC, KLF4 at MOI 5:5:3), achieving robust pluripotency with normal morphology, expression of OCT4/SOX2/TRA-1–60/NANOG/SSEA4, trilineage differentiation potential (PAX6/SOX1 ectoderm, GATA4/FOXA2 endoderm, ACTA2/TBXT mesoderm), normal karyotypes (46,XY/46,XX), complete Sendai vector clearance by passage 10, identical STR profiles to parental fibroblasts, and mycoplasma-free status [102]. These healthy control lines provide essential isogenic baselines for LFS and other patient-derived iPSC models, enabling precise dissection of disease-specific phenotypes while demonstrating the scalability and genetic fidelity of Sendai virus reprogramming for oncology applications. Advancing to PBMC sources, a 2024 study reported creation of iPSCs-lines from LFS patients with TP53 mutation, validating pluripotency markers, three-germ-layer differentiation, teratomas, and stable genotypes to model haploinsufficiency across lineages. Sun et al. produced two Sendai virus-derived hiPSC lines (SCVIi104-A, c.743G > A; SCVIi105-A, c.467G > A) from LFS PBMCs, confirming pluripotency (OCT4, SOX2, NANOG immunofluorescence/RT-qPCR), trilineage potential, teratomas, normal karyotypes, Sendai clearance, STR identity, and retained TP53 mutations, establishing expandable platforms for LFS sarcoma/breast modeling [103]. Collectively, these chronologically evolving hiPSC approaches from functional OB sarcoma genesis models to standardized PBMC-derived repositories provide renewable, mutation-faithful systems that bypass primary cell limitations, enabling precise dissection of TP53-driven early events in LFS oncogenesis and high-throughput therapeutic screening in sarcoma-susceptible lineages.

Another bottom-up approach inserts specific oncogenic mutations into otherwise normal hiPSCs using CRISPR/Cas9 and associated base-editing tools to create isogenic cancer models that replicate specific tumor-evolution pathways [104]. In the sequential CRISPR hiPSC model of myeloid disease, stepwise introduction of mutations in genes such as epigenetic or splicing regulators together with RAS-pathway lesions produced a series of isogenic hematopoietic derivatives that recapitulated clonal hematopoiesis, MDS-like states, and fully leukemic phenotypes, with later combinations showing increased self-renewal, impaired differentiation, and selective engraftment advantages in vivo [104]. Multi-allelic base-editing models in epithelial cells and organoids similarly demonstrated that defined sets of canonical tumor suppressor alterations (for example APC and TP53) combined with RTK/RAS-pathway mutations (such as KRAS or PIK3CA) generate graded shifts in proliferation, differentiation, and drug responses, indicating that specific mutational constellations and their timing govern whether clones remain pre-malignant or progress to aggressive, therapy-resistant disease [105]. Complementary clonal and phylogenetic analyses of human AML samples further show that early “landscaping” mutations in chromatin/epigenetic regulators (e.g., DNMT3A, TET2, IDH1/2, ASXL1) establish persistent pre-leukemic stem cell compartments, whereas later “proliferative” hits in signaling genes (FLT3, NRAS, KRAS) drive clonal sweeps, genomic instability, and relapse after therapy, reinforcing the principle that mutation class and order together dictate clonal dominance, transcriptional programs, and malignant traits such as enhanced self-renewal and treatment resistance [106].

These systems have demonstrated that the sequence and combination of mutations (e.g., chromatin regulators and signaling genes in myeloid disease, or canonical tumor suppressors and RTK pathway components in solid tumors) have a critical role in dictating clonal dominance, transcriptional condition, and the development of malignant phenotypes such as enhanced self-renewal, genomic instability, and drug resistance. Taken together, these reports indicate that engineered hiPSC models can recapitulate early pre-malignant and malignant transitions in a controlled genetic and developmental background, which allows dissection of cancer predisposition alleles, context-specific DNA damage responses, and genotype-specific drug sensitivities in a manner that complements patient-derived tumor iPSCs.

To further illustrate how hiPSC platforms can uncover post-transcriptional mechanisms beyond canonical genomic drivers, a hereditary retinoblastoma patient-derived iPSC model revealed that RB1 loss promotes bone malignancy-associated phenotypes in part through aberrant spliceosome function [107]. Combined transcriptomic and regulatory studies presented in the context of hiPSC-derived osteoblasts revealed that perturbation of the pRB axis is associated with widespread spliceosomal gene program upregulation, including pRB/E2F3a-regulated spliceosome components at promoters and enhancers, and thus related changes in RB pathways to spliceosome rewiring in the face of oncogenic stressors [107]. Notably, pharmacologic spliceosome inhibition induced systematic intron retention and reduced proliferative and tumorigenic phenotypes in RB1-deficient models, suggesting the spliceosome machinery as a potentially targetable vulnerability in RB1-deficient bone malignancies, extending beyond its canonical role in cell-cycle deregulation [107].

In addition to the creation of genome-edited hiPSC cancer lines, hiPSC-based organoid and so-called assembloid platform provide a renewable three-dimensional system in which engineered genotypes can be implemented in lineage-relevant tissues and a reconstituted microenvironment. These methods can recapitulate the interactions of defined oncogenic programs with stromal, vascular, and immune-like compartments (through co-differentiation or co-culture), as well as facilitate systematic interrogation of tumor-microenvironment crosstalk, drug transport, drug-penetration challenges, and immunotherapy-relevant behaviors in three-dimensional (3D) microenvironments. Simultaneously, iPSC-derived organoids have the potential to support scalable perturbation screens, including drug and functional genomic screens, in well-established, lineage-resolved backgrounds. These systems are, however, still constrained by maturation or state fidelity, standardization, and incomplete immune-stromal representation, which encourage benchmarking against primary tumor data, and where needed, complementary platforms, including microfluidics- and organ-on-a-chip-based systems.

### Advantages of hiPSC-derived cancer platforms over conventional models

The major advantage of hiPSC-based cancer models is their capacity for long-term self-renewal and scalability. When established under rigorous quality-control standards, these models can be expanded to generate consistent, reproducible, and standardized cellular resources for long-term mechanistic investigation. However, extended passaging can introduce genomic instability and epigenetic drift, requiring genomic surveillance, epigenetic monitoring, and tightly standardized culture conditions. Notably, reprogramming approaches maintain the genetic modifications of the parent tumor, which provides a fresh platform upon which the mutational landscape of the patient is truly reflected [108]. The other notable benefit is that they enable one to capture patient specific heterogeneity. Since the hiPSCs are purified out of individual tumors, they do not only maintain genetic but also epigenetic diversity, a characteristic that is extremely important in therapeutic response [19]. By contrast, cell lines and PDOs that have been passaged many times are more likely to be drawn towards more homogenous transcriptional states, hiding clinically relevant variability. Moreover, hiPSCs inherently permit the production of isogenic controls, permitting a direct comparison of mutant and fixed genotypes in the identical genetic background, and permit a fine-scale analysis of the effect of certain mutations on therapeutic susceptibility.

Besides these strong underpinnings, hiPSC-derived cancer models provide an opportunity of longitudinal studies of therapeutic resistance. Their ability to self-renew means that researchers can study evolutionary processes over long periods without genetic drift seen in immortalized cell lines and without exhaustion observed in PDO cultures, and that stromal fibroblasts, endothelial cells, and innate or adaptive immune populations may be co-differentiated or co-cultured into different complex tumor ecosystems with high fidelity. This allows the comprehensive modeling of interactions between tumors and immune-stromal interaction, which are becoming a greater determinant of treatment outcome. In addition, hiPSCs can provide a very tractable platform to perform large-scale functional genomic screens, such as CRISPR/Cas9, RNA interference, and base-editing techniques, and in well-defined isogenic backgrounds. They are also scalable and homogeneous, and thus, make hiPSC-derived derivatives highly suitable for a large-scale drug testing, something that cannot be done with PDXs because of cost and throughput constraints, or with PDOs because of poor culture performance. Combinations of these strengths make hiPSC-derived cancer models a highly applicable and confident next-generation platform to find and evolve discovery in and develop precision therapies.

## Applications of hiPSC-derived platforms in precision oncology

**Fig. 4:**
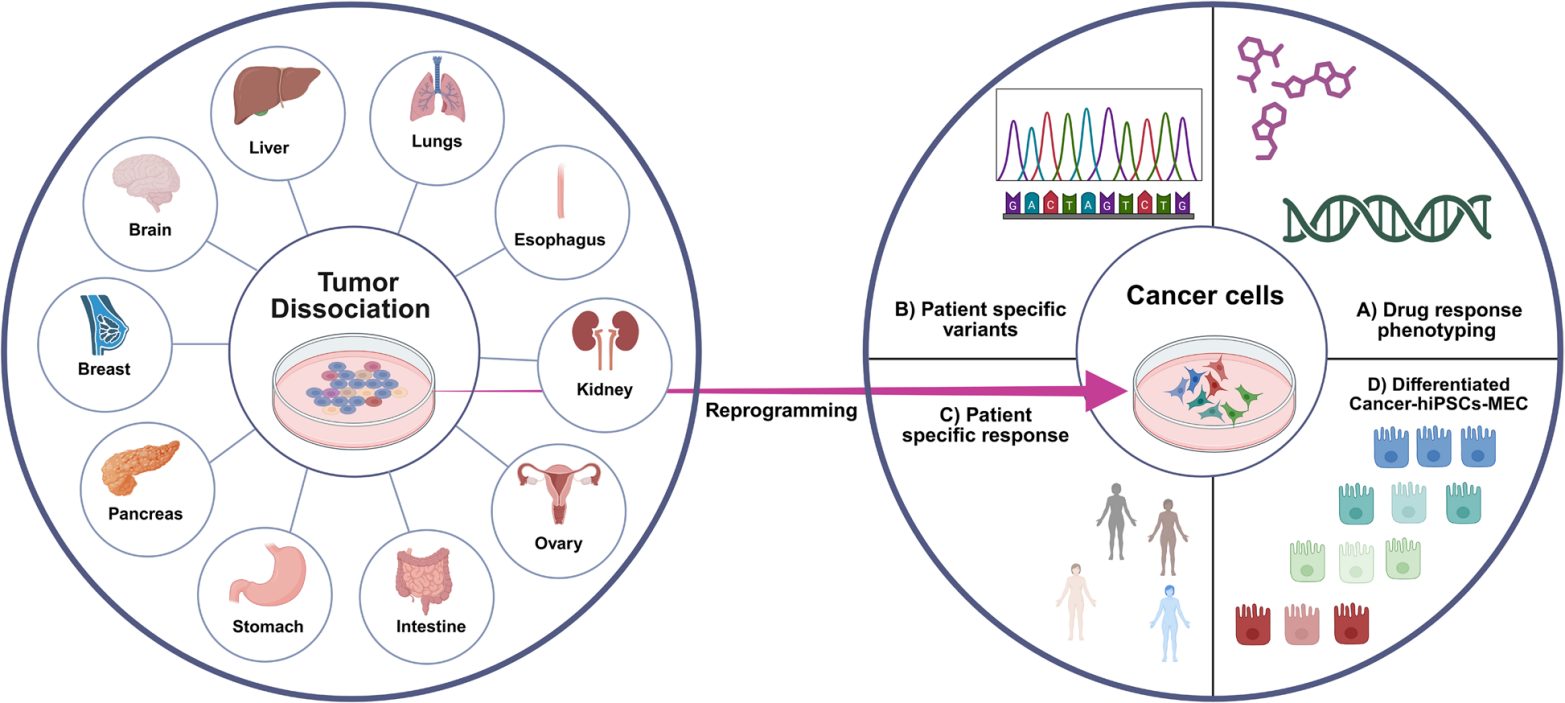
Application of hiPSC derived cancer models in precision oncology: iPSCs: Induced Pluripotent Stem Cells; MMC: Mammary Epithelial Cells

The applications of hiPSC-based cancer models are not only limited to basic research but also useful in practical use in precision oncology. The patient specificity, possibility of unlimited renewal and compatibility with isogenic control all render hiPSCs exceptionally applicable in the case of those uses that demand in-depth reproducibility. Here, we emphasize the applications of hiPSCs-derived tumor models in (i) predicting individual patient drug response, (ii) modeling therapeutic resistance, (iii) rational combination regimens, and (iv) immuno-oncology tumor-immune interactions, and (v) prediction of individual- and tissue-specific toxic responses to cancer therapy. Collectively, these use cases illustrate the potential of hiPSC-derived cancer models to narrow the gap between preclinical modeling and clinical decision-making (Fig. 4; Table 4). Across these application areas, hiPSC-derived organoid systems are emerging as a particularly powerful 3D platform for therapy testing because they enable lineage-resolved, renewable modeling of tumor-relevant tissue contexts, and can be integrated with immune and stromal components or microenvironmental engineering strategies to more effectively capture clinical resistance mechanisms and drug transport phenomena.

**Table 4:** Key original research studies applying hiPSC-based cancer models

Cancer type/key mutation(s)	Approach/Model type	Model details	Key findings/therapeutic readout	References
Primary breast cancer-patient/subclone-specific BRCA1/2 variants	Tumor → reprogramming → hiPSC	Reprogrammed primary breast tumor cells into hiPSCs; differentiated into mammary epithelial cells and organoids	BC-hiPSCs retained patient & subclone mutations; BRCA1/2-harboring lines were PARPi-sensitive; CRISPR correction of BRCA1 reversed PARPi sensitivity → causal genotype → phenotype demonstration	[19]
Myeloid malignancy progression (CH → MDS → AML); stepwise driver acquisition	Sequential CRISPR/Cas9 editing of a normal human iPSC line → hematopoietic differentiation → staged disease modeling	Stepwise editing of a normal, well-characterized iPSC line to include mutations that cause myeloid disease (e.g., epigenetic/splicing regulator lesions combined with RAS-pathway hits), and then differentiated into hematopoietic derivatives to represent clonal evolution between pre-malignant and malignant states	Incremental mutation acquisition produced a chain of isogenic hematopoietic derivatives that recapitulated graded phenotypes consistent with clonal hematopoiesis/MDS-like states and leukemic phenotypes and allowed measurement of the effects of mutations on the order of acquisition and combination on self-renewal, differentiation, and malignant progression	[90]
Li-Fraumeni syndrome/mutant TP53; iPSC-derived astrocyte	LFS iPSC-derived lineage model + epigenetic/epitranscriptomic mechanistic dissection and functional inhibition	LFS iPSC-derived astrocytes were used to test how mutant p53 initiates neoplastic transformation through coupling chromatin regulation to m6A-dependent RNA fate control	Mutant p53 (not WT p53) interacted with SVIL and recruited MLL1/KMT2A to enhance H3K4me3 at the YTHDF2 promoter and boosted YTHDF2 and decay of m6A-tagged tumor-suppressive transcripts (including CDKN2B and SPOCK2). YTHDF2 genetic depletion or pharmacologic inhibition of the MLL1 complex suppressed mutant p53-induced neoplastic phenotypes, and this epitranscriptomic axis is actionable	[101]
Hereditary retinoblastoma/RB1 loss; osteosarcoma-relevant bone malignancy mechanism	Patient-derived hereditary RB iPSC model → osteoblast/bone lineage modeling and functional perturbation	iPSC model of hereditary RB was used to determine how RB1 deficiency induces phenotypes of bone malignancy and to map downstream regulatory programs beyond the canonical deregulation of the cell cycle	RB1 loss was associated with upregulated spliceosome programs (involving pRB/E2F3a-mediated regulation of spliceosomal genes). Broad splicing disruption (including intron retention) and inhibition of the proliferative/tumorigenic phenotype by pharmacologic spliceosome inhibition supported the spliceosome as an actionable vulnerability in pRB-deficient cancers	[107]
Breast cancer -germline BRCA1	Patient iPSCs → lineage differentiation	Generated mammary-like cells from patient iPSCs carrying BRCA1 mutation	Discovered S100P acts as a mediator of cancer stemness in the BRCA1 setting; genotype-stemness phenotype associations with therapeutic implications	[109]
RET-rearranged NSCLC (RET^C634Y)	Patient iPSC reprogramming → lineage differentiation	iPSCs derived from RET-mutant patient; differentiated to lung progenitors	RET-mutant iPSC-derived lung progenitors displayed sensitivity to RET inhibitor (pralsetinib), recapitulating genotype-matched drug vulnerability	[110]
Mutations in oncogenes (PTPN11, TP53)	Malignant cell → reprogramming → hiPSC Patient iPSC reprogramming → lineage differentiation	Malignant cell → reprogramming → hiPSC” and “Patient iPSC reprogramming → lineage differentiation	Engineered hiPSC progenitors served as “synthetic cancer” models for in-vitro modeling of human malignancy; and investigate the underlying pathological mechanisms	[17, 103]
iPSC-derived tumors (multiple lineages)/autologous tumor-immune context	Autologous humanized mouse model	The human IPS-derived tumor cells were transplanted into donor-matched, autologous mice to demonstrate that in vivo donor-matched profiling and perturbation of tumor-immune interactions across different iPSC-derived tumor settings were feasible	The autologous immune cells were introduced into iPSC-derived tumors and occasionally led to tumor rejection. These tumors had exhausted T cells that expressed PD-1 and TIM-3, and an increased number of Tregs. Targeting the immune checkpoint with anti-PD-1 enhanced immune cell infiltration but yielded limited antitumor efficacy. This strategy offers an example of investigating and testing cart tumor-immune interactions in a mismatched but related system	[111]

*AML* Acute Myeloid Leukemia, *BRCA1* BReast CAncer gene 1, *CDKN2B* Cyclin-dependent kinase 4 inhibitor B, *CRISPR* Clustered Regularly Interspaced Short Palindromic Repeats, *Cas9* CRISPR-associated protein 9, *H3K4me3* trimethylation of histone H3 at lysine 4, *LFS* Li-Fraumeni syndrome, *KMT2A* Lysine-specific Methyltransferase 2 A, *iPSC* Induced pluripotent stem cell, *NSCLC* Non-small cell lung cancer, *PD-1* Programmed Cell Death Protein, *PTPN11* Protein Tyrosine Phosphatase Non-Receptor Type 11, *PARPis* Poly (ADP-ribose) polymerase inhibitors, *RB* Retinoblastomas, *Tregs* Regulatory T cells, *TIM3* T-cell immunoglobulin and mucin-domain containing-3, *TP53* Tumor Protein p53, *YTHDF2* N6-methyladenosine RNA binding protein 2

### hiPSC-derived models for precision drug response

The prediction of individual patient- and genotype-specific drug responses in lineage-relevant cellular environments can be among the most extensive uses of hiPSC-based cancer models. As hiPSCs maintain patient germline backgrounds, can be engineered with oncogenic variants, and can be directed to cell-of-origin lineages, direct testing of specific therapies under genetically controlled conditions can be performed using hiPSCs. In combination with isogenic mutation-corrected or mutation-introduced controls, such systems can be causally attributed to individual variants in drug sensitivity. This is demonstrated through representative studies in BRCA1-mutant patient-derived hiPSC mammary models and oncogene-rearranged systems. For example, Liu et al. generated germline BRCA1-mutant patient-derived hiPSCs, differentiating them with > 90% efficiency into CK5/CK8-positive mammary epithelial cells/organoids using stepwise growth factor protocols, and created isogenic BRCA1-restored controls to isolate mutation-specific effects. BRCA1 loss drove cancer stemness through S100P upregulation via direct promoter regulation, activating RAGE/NF-κB signaling (OCT4/SOX2/NANOG elevation) while simultaneously inducing replication stress, HRD phenotypes, and synthetic lethality with PARP inhibitors, demonstrating that hiPSC platforms can dissect both stemness circuits and DDR vulnerabilities difficult to resolve in conventional cell lines. These dual insights position BRCA1-mutant hiPSC mammary models as discovery engines for mutation-specific combination strategies pairing PARP inhibitors with stemness or additional targeting agents in key DDR pathways such as Ataxia Telangiectasia and Rad3-Related Kinase (ATR) and G2 checkpoint kinase (WEE1) within defined genetic backgrounds [109]. A conceptually similar approach was taken by Marcoux et al., who generated lung progenitor cells (LPCs) from patient-derived iPSCs carrying RET rearrangements to model NSCLC, showing that these RET-rearranged LPCs maintained the patient-specific oncogenic fusion, exhibited aberrant downstream signaling consistent with RET activation, and provided a platform to test RET-targeted therapies in a controlled, isogenic developmental context [110]. Taken together, these studies show that hiPSC-derived cancer models can mechanistically couple patient genotype to drug-response phenotype in controlled, isogenic, and lineage-appropriate systems. By enabling genome editing, matched controls, and scalable subclone derivation, hiPSC platforms provide more causally interpretable drug response modeling than conventional cell lines and complement PDO and PDX approaches in precision oncology.

### Modeling therapeutic resistance

hiPSC cancer models can effectively be applied to study drug resistance mechanisms due to their capacity to combine renewable scaling of identical genotypes, lineage-guided differentiation into subsequent disease-relevant cell types and causal genetic testing via isogenic genome editing. Collectively, these functions facilitate the reconstructive and analytical control of resistance-related conditions that are hard to cure in heterogeneous preclinical systems. The hiPSC-based resistance modeling is usually pursued in modern practice in accordance with a few complementary approaches [112, 113]. One establishes matched model sets by cloning patient tumor cells into hiPSCs and/or assembles engineered isogenic panels where a given variant is introduced, corrected and/or reverted in a common genetic background. This design reduces confounding genomic variation and is useful in causal inference. Second, hiPSCs are differentiated into lineage-relevant cell types that reflect the suspected cell of origin e.g. mammary epithelial, lung progenitor or hematopoietic derivatives such that drug response and resistance states are studied in the relevant developmental and transcriptional states. Third, longitudinal or stepwise exposure to a drug can be done on lineage-matched cultures grown as two-dimensional cultures or organoid systems, and resistant populations can be selected and expanded with matched untreated controls. Fourth, the interrogation of resistance mechanisms is achieved through parental versus drug-selected derivatives comparison based on functional assays and multi-omics profiling to differentiate pre-existing and adaptive resistance subclones. Normal reads are pathway activity, DNA damage and repair competency, and transitions in lineage states. Lastly, to test causality of candidate resistance determinants, genome editing and isogenic perturbation can be used, and rational combination strategies can be assessed to prevent or overcome therapeutic escape. A representative example is the breast cancer hiPSC platform described by Weddle et al. in which primary breast cancer cells from nine patients across major molecular subtypes can be efficiently reprogrammed into bona fide breast cancer-derived hiPSCs (BC-hiPSCs) that retain both germline susceptibility variants (including BRCA1 and BRCA2) and patient- and subclone-specific somatic mutations, thereby capturing intratumoral genomic heterogeneity in a renewable pluripotent resource [19]. The authors then establish a robust differentiation protocol that drives BC-hiPSCs into luminal-biased mammary epithelial cells (BC-hiPSC-MECs) and mammary-like organoids, which closely mirror the gene expression profiles of the originating primary tumor cells, confirming restoration of a breast epithelial state suitable for disease and drug-response modeling [19]. Using this platform, they show that BC-hiPSC-MECs harboring pathogenic BRCA1 or BRCA2 variants exhibit pronounced, selective hypersensitivity to PARP inhibitors (olaparib and talazoparib), while BRCA-wildtype lines remain resistant and all lines respond similarly to doxorubicin, firmly linking homologous recombination status to PARP inhibitor response in a patient-specific context. Mechanistic assays reveal that BRCA1-mutant BC-hiPSC-MECs accumulate γH2AX foci but fail to form RAD51 foci after PARP inhibition, and that CRISPR-mediated BRCA1 knockout in control hiPSCs replicates both the drug sensitivity and RAD51 defect, directly implicating BRCA1 loss in impaired homology-directed repair and synthetic lethality with PARP inhibition [19]. Finally, precise CRISPR introduction and correction of the patient’s BRCA1 c.68_69delAG founder variant demonstrate that this single allele is necessary and sufficient for PARP inhibitor hypersensitivity and RAD51 repair defects, establishing BC-hiPSCs as a powerful, genetically tractable preclinical model to dissect patient- and variant-specific determinants of PARP inhibitor response in breast cancer. This may pave the way for future work that will systematically map HR-pathway variants, sub clonal genotypes, and combination therapies in a scalable, lineage-appropriate platform to guide truly individualized PARP inhibitor use [19]. Together, these findings establish breast cancer-derived hiPSC models as genetically tractable systems for dissecting variant-specific determinants of PARP inhibitor response and for guiding mechanism-based combination strategies in lineage-relevant platforms. Collectively, these findings demonstrate that hiPSC systems can support resistance-relevant, genotype-linked therapeutic assessment on controlled genetic backgrounds. Since hiPSC cancer systems enable isogenic editing, lineage regulation, and scalable derivation of subclones, they enable more direct causal dissection of resistance determinants as compared to standard models based on cell lines, in addition to complementing PDO- and PDX-based studies.

### Rational design of combination therapies

Single-agent approaches show limited efficacy and durability in cancer treatment and combination therapies are increasingly necessary given redundancy of pathways, rewiring of adaptive signaling, and clonal heterogeneity. With selective drug pressure, tumors often induce countermeasures to checkpoint and repair mechanisms or increase the size of already resistant subclones, resulting in resistance to therapy. Consequently, a mechanistically-based combination of drugs is employed to gain better pathway suppression and enhance the persistence of anticancer responses. Such synthetic lethality based on strategies targeting complementary survival pathways have well been developed as a concept and a clinical framework e.g., in the literature on DNA damage response (DDR)-targeted therapies [114, 115]. This is exemplified by the case of DDR-deficient tumors, wherein PARP inhibitors exhibit high activity at baseline, but potentially fail due to replication-stress adaptation and checkpoint compensation. Various preclinical data suggest that regimens comprising PARP inhibitors, when used in combination with checkpoint or replication-stress pathway inhibitors, such as ATR or WEE1 inhibitors, can be utilized to obtain an enhanced level of cytotoxicity, which is a mechanistic argument that justifies rational drug combination design [116].

In this context, hiPSC-based cancer models are an engineered source of genetically regulated and lineage-resolved platforms for the development of mechanism-based drug combinations. Since they can be utilized to conduct isogenic comparisons, to repair mutations, and to perturb specific pathways, they can be used to test drug-drug interactions in matched genetic backgrounds with minimal confounding genomic variation. Lineage-guided and mutation-defined hiPSC derivatives also enable the combination responses to be evaluated in the relevant cell-of-origin context. The most recent hiPSC-based cancer modeling studies show that genotype-stratified drug response testing and causal variant validation are feasible and that these platforms can be used to test drug combination strategies [19, 109, 110]. The hiPSC systems minimize the background genetic drift and permit matched genome-editing manipulations as compared to conventional cancer cell lines. They easily facilitate scalable isogenic perturbation and parallel lineage-specific testing compared to PDO and PDX models. Combined, these properties enable hiPSC-derived cancer platforms to be potent and powerful complementary tools to generate mechanism-directed combination therapy for precision oncology.

### Immuno-oncology modeling

hiPSC-based models are now being expanded into the tumor-immune interaction paradigm, as recent reports include engineered iPSC-derived tumor-immune models, and autologous iPSC-derived immune effector systems [114, 117, 118]. Antigen presentation, immune infiltration and exhaustion, and the development of immunosuppressive programs are all regulated by tumor-immune crosstalk that dictates the durability of tumor responses to checkpoint blockade and other immunotherapies [119]. One of the main benefits of hiPSC platforms is that it is possible to create genetically compatible tumor-immune systems. Since hiPSCs can produce tumor-lineage and immune effector in the same donor genetic backgrounds, they provide causal analysis of immune recognition and escape pathways based on isogenic perturbation. A study by Moquin-Beaudry et al. provides an example of how hiPSC-derived platforms can be used to explore tumor-immune interactions systematically and in vivo using autologous humanized mouse models, in which tumor-derived platforms are used alongside matched human immune systems [111]. Concurrently, the speed of development in iPSC-derived immune effectors, especially iPSC-NK and engineered CAR-iNK/CAR-iT systems offers scalable and standardized immune components, which can be implemented in co-culture systems to investigate cytotoxicity, immune suppression, and candidate immunotherapy combinations [117]. Even though the field is still under development and this has been hindered by immune maturation status, microenvironmental complexity, and long-term co-evolution, preliminary research indicates that patient-aligned immunotherapy testing, and mechanistic research of tumor immune evasion can be achieved with hiPSC-derived systems. Relative to traditional co-culture models based on unsurpassed immune donors, hiPSC-based platforms provide a route to scalable, isogenic and lineage resolved tumor immune models to complement PDO/PDX systems in the development of mechanistic immuno-oncology and integration-based combination strategies. In addition, tumor organoid co-culture formats, particularly when paired with iPSC-derived immune effectors, provide a scalable framework to quantify infiltration, cytotoxicity, and immune escape programs in 3D microenvironments.

### Individual and tissue-specific toxicity prediction of cancer therapy (hiPSC-based safety pharmacology)

Unanticipated toxicity is the leading cause of clinical trial failure in late stages, of dose constraint, and of post-marketing drug loss in oncology. Safety liabilities like cardiotoxicity, hepatotoxicity, nephrotoxicity, and neurotoxicity raise development costs substantially and add to high attrition rates of anticancer drug pipelines. The large-scale analysis indicates that the majority of oncology drugs do not succeed in the clinical development process, and the most frequent reasons of the attrition are safety and efficacy concerns [120]. Traditional preclinical safety model types commonly use transformed cell lines or other non-human systems and are often not able to reflect patient-specific genetic predisposition and tissue-selective vulnerability. Such constraints have led to rising interest in human genotype-resolved multi-tissue model systems of predictive toxicity, represented by hiPSC-based models due to the indefinite renewability and multi-tissue-like differentiation characteristics of patient-derived hiPSC-CMs [121]. hiPSC-CMs have been extensively shown to predict proarrhythmic and cardiotoxic drug effects and are integrated into contemporary safety pharmacology paradigms including the Comprehensive in Vitro Proarrhythmia Assay (CiPA). In the same way, hiPSC-derived hepatocyte-like cells and liver organoid systems have been utilized to simulate drug-induced liver injury and inter-individual hepatotoxic vulnerability [122]. hiPSC-derived renal proximal tubule and kidney organoids can be used to assess kidney toxicity through injury responses to nephrotoxic drugs [123]. Neurotoxicity and developmental neurotoxicity can be assessed using hiPSC-derived neural lineage systems and 3D neural cultures with functional readouts.

One way the hiPSC-based toxicity scanning can be practically implemented is by building multi-tissue testing panels using patient-derived or engineered isogenic hiPSC lines. There are several lines separated into cardiomyocyte, hepatic, renal and neural derivatives and subjected to candidate anticancer agent and drug combinations in acute and repeated dose regimens. Toxicity is measured with lineage-specific functional toxicity assays such as electrophysiology of arrhythmia risk, liver injury biomarkers, renal injury biomarkers, and neural functional biomarkers that allow early detection of dose limiting toxicities and mechanism-based safety liabilities within a genotype matched system [80, 124]. Notably, causal testing of toxicity mechanisms using hiPSC systems is also possible by isogenic introduction of genome edits whereby toxicity phenotypes can be directly linked to a particular susceptibility allele by introducing or repairing variants in matched backgrounds. Inter-individual variability in drug responses may also be assessed using population-scale iPSC panels [125].

## Challenges of hiPSC-derived models of cancer

The hiPSC-based cancer models are currently of great interest due to their potential to be utilized as promising platforms for patient-specific disease modeling and precision oncology. However, these models are still technically challenging, and they are utilized by a comparatively limited number of specialized research teams. These systems have regenerable, patient-specific frameworks, but the general implementation of them in oncology studies is restricted by technical, biological, and ethical issues that impact reliability, reproducibility, and translational preparedness. It will be necessary to overcome these challenges, through technological innovation in reprogramming, differentiation, quality control and governance systems that will enable hiPSC-derived cancer models to achieve their full potential in the drug discovery, resistance mapping, and personalized therapy.

### Technical hurdles in reprogramming and differentiation

Reprogramming efficiency and fidelity can vary significantly by tissue source, tumor type, and mutational background, and malignant cell reprogramming is frequently highly inefficient and heterogeneous [126]. Oncogenic mutations such as TP53, MYC deregulation, and complex karyotypes may disrupt complete reprogramming, may select subclones, or may trap cells in intermediate reprogrammed states, making downstream cancer modeling more difficult [126, 127]. Moreover, differentiation into tumor-relevant lineages often yields mixed populations of variable maturity and identity, making the normalization of assays and cross-study comparisons across lines, protocols, and laboratories challenging. Further streamlining of the lineage-directed differentiation and maturation procedures will thus be essential to enhance model consistency and functional interpretability [126].

### Maturation state of hiPSC-derived tissues: implications for adult-onset cancer modeling

A recognized limitation of most hiPSC-derived tissues is incomplete maturation, as differentiated tissues can retain fetal-like characteristics and lack the transcriptional, metabolic, and functional features of their in vivo counterparts [128]. This limitation is particularly relevant to cancer modeling, especially for adult-onset malignancies (e.g., epithelial carcinomas), because developmental state can modulate signaling dependencies, cell-state composition, micro-environmental interactions, and basal stress-response circuitry, which can subsequently affect observed drug-response phenotypes. Accordingly, therapeutic evaluation in hiPSC-based cancer models should explicitly account for maturation state and incorporate benchmarking strategies, such as comparisons with primary adult tumor datasets and well-annotated PDO/PDX response profiles linked to clinical outcomes. Where feasible, maturation-enhancing approaches, including extended culture, 3D culture systems, organoid-based differentiation, metabolic modulation, nanopatterning of culture surfaces, electrical stimulation, and co-culture with hiPSC-derived complementary cell types, are increasingly used to promote adult-like phenotypes and improve translational interpretability [129–132].

### Genetic and epigenetic fidelity, and biosafety risks

While hiPSC-based cancer models can maintain patient-specific driver mutations, reprogramming causes global epigenetic remodeling and partial resetting of epigenetic memory can change disease phenotypes [133, 134]. When glioblastoma-derived hiPSCs are used, it has been demonstrated that the tumor-associated patterns of methylation can be re-established in part following differentiation, but not always restore tumor behavior, suggesting that epigenetic reconfiguration can alter tumorigenic potential compared to the parenting tumor [135]. Genomic instability, such as aneuploidy, copy-number variation, and point mutations, is also linked to long-term expansion of hiPSC and can be beneficial to growth, whereas impairing disease fidelity and presenting safety risks [136, 137]. Besides, residual undifferentiated cells are associated with the risk of teratoma, and mutations gained during the culture could present unwanted malignant characteristics. These dangers lead to the importance of strict genomic, epigenomic, and functional quality control methods prior to the application of hiPSC-derived cancer models for mechanistic or translational inferences [138].

### Reproducibility and standardization across laboratories

There are significant differences in the phenotype and biomarker outputs across different laboratories because of protocol variability, hiPSC derivation, culture conditions, differentiation strategies, and assay readouts [113]. The same problems, such as batch effects, dependence on the matrix, variability in media composition, and scoring inconsistencies can impair comparability and translational interpretation, have been observed with patient-derived organoid systems [139]. The standardized derivation and authentication processes, reference differentiation protocols, assay benchmarking, curated biobanks, and coordinated multi-site validation initiatives using international consortia will need to be relied on to drive advancement across the field.

### Ethical and regulatory considerations

hiPSC-based cancer models utilize recognizable patient tissues and often include significant genetic manipulation, which also introduces significant ethical and regulatory implications regarding informed consent, long-term storage and reuse, sharing of genomic data as well as the handling of genetically unstable or tumorigenic lines [140]. In most jurisdictions genome-edited hiPSC systems are regulated by both human subject research and those dealing with genetically modified organism’s systems, necessitating layered regulation and governance [141]. It will take responsible translational integration to include standardized language of consent covering extensive future use, transparent data management, and harmonized international regulatory frameworks to provide safe, ethical, and socially acceptable use.

## Conclusion and future perspectives

hiPSC-derived cancer models are becoming potent experimental platforms for precision cancer therapy with renewable patient genotypes, lineage-controlled differentiation, and isogenic engineering in human cellular systems. Combined with multi-omics profiling, lineage tracing, sophisticated imaging, microengineered culture systems, and computational analytics, these models are no longer simply a reflection of a disease but a dynamic, mechanism-resolved, and patient-aligned translational tool. These convergent strategies together enhance the connection between molecular discovery and therapeutic decision-making.

One potential avenue of application of hiPSC-based models is the mapping of longitudinal resistance evolution. For example, hiPSC systems can be scaled to allow genetic backgrounds to undergo controlled subclone derivation and to enable potential tracking of therapy adaptation during chronic drug selection [19]. Combining serial drug exposure with single-cell profiling, CRISPR perturbation screens, and multi-omics analysis can identify mutation-specific networks of resistance and dynamic biomarkers that are challenging to measure in passage-limited PDO or PDX models. Lineage-tracing and barcoding CRISPR-based methods also enable direct tracking of clonal dynamics and the development of resistance to therapeutic pressure [142–144].

Microengineered and spatially resolved platforms have the potential to further increase model fidelity. A combination of hiPSC-derived tumor and stromal subunits with organ-on-a-chip and perfusable microphysiological environments enables a more realistic model of drug exposure, immune infiltration, and tumor-stroma interactions [145]. Simultaneously, live-cell imaging and spatial transcriptomic technologies allow for mapping state changes induced by therapy and microenvironmental gradients present in hiPSC-derived tumor systems in high-resolution [146, 147].

Immuno-oncology modeling and tumor-microenvironment reconstruction matched on a patient basis is an active, yet emerging direction [148, 149]. Generation of tumors, immune, and stromal lineages that share hiPSC genetic backgrounds and can be studied using three-dimensional microfluidic and spatial immunomics may permit the localized study of immune evasion and microenvironment-mediated mechanisms of resistance. Biomaterials, nanostructured scaffolds, and tumor-mimicking matrices are expected to be further improved in the future to enhance three-dimensional niche modeling and drug penetration measures [150, 151].

Computational modeling and artificial intelligence will be used increasingly as hiPSC platforms generate high-dimensional, multimodal data. Omics, imaging, and functional response data can be incorporated to aid in predicting sensitivity, resistance development, and rational combination of drugs in known genetic settings [152]. Future modeling frameworks utilizing co-clinical and patient-matched modeling can potentially enable the prediction of drug response in prospective studies and the optimization of regimens in individuals.

To conclude, hiPSC-derived models of cancer must be considered high-resolution complements to PDO and PDX models, not substitutes. They are highly renewable, genetically manipulable, can be tailored to specific lineages, are compatible with new analytical technologies, and have high long-term potential. As technical perfection, standardization, and responsive governance are maintained, hiPSC-based cancer modeling are expected to become a significant part of translational oncology and precision medicine for patients.

## Data Availability

No datasets were generated or analysed during the current study.

## Abbreviations

ADCs: Antibody–drug conjugates
ADCC: Antibody-dependent cellular cytotoxicity
Akt: Protein Kinase B
BRCA1: BReast CAncer gene 1
CTCs: Cancer circulating tumor cells
CAM: Chorioallantoic membrane
CNAs: Copy number aberrations
DDR: DNA damage response inhibitors
EGFR: Epidermal growth factor receptor
hiPSC: Human induced pluripotent stem cell
HGSOC: High-grade serous ovarian cancer
HER2: Human epidermal growth factor receptor 2
HIF1A: Hypoxia-inducible factor 1 alpha
iPSC-MCs: Mammary-like epithelial cells
mAbs: Monoclonal antibodies
MAPK: Mitogen-activated protein kinase
ML: Machine learning
NSCLC: Non-small cell lung cancer
LFS: Li-Fraumeni syndrome
DDRis: DNA damage response inhibitors
PBMC: Peripheral blood mononuclear cells
PD-1: Programmed cell death protein 1
PD-L1: Programmed death-ligand 1
PARPis: Poly (ADP-ribose) polymerase inhibitors
PDOs: Patient-derived organoids
PDXs: Patient-derived xenografts
PDOX: PDO xenografts
PitNETs: Pituitary neuroendocrine tumor
PI3K: Phosphoinositide 3-Kinase
RET: Rearranged during Transfection
RTK: Receptor Tyrosine Kinase
TNBCs: Triple Negative Breast Cancer Cells
